# Posterior Epidural Migration of Lumbar Intervertebral Disc Fragment Mimicking an Epidural Mass

**DOI:** 10.7759/cureus.47522

**Published:** 2023-10-23

**Authors:** Ayesha A Ansari, Paresh Korde, Syed Yasir Afaque, Shraddha Sawhney, Naman Mishra

**Affiliations:** 1 Neurosurgery, Jawaharlal Nehru Medical College, Datta Meghe Institute of Higher Education and Research, Wardha, IND; 2 Oncology, Nottingham University Hospital National Health Services Trust, Nottingham, GBR

**Keywords:** epidural mass, lumbar intervertebral discs, migration, epidural, posterior

## Abstract

Acute and chronic lower back pain can be commonly caused by intervertebral disc prolapse. This prolapse usually occurs in the dorsal direction and towards the anterior epidural space. In extremely rare cases, this migration/herniation can be seen approaching the posterior epidural space. One such rare instance has been recorded and described in our patient, a 53-year-old with a history of hypertension who presented with persistent lower back pain, radicular in nature, and recent acute aggravation, leading to mobility impairment. The patient experienced numbness in the lower limbs, urinary incontinence, and irregular bowel movements. Sensory deficits were noted along the L3 dermatome.

The patient underwent an L3 laminectomy, revealing extruded disk fragments causing the compression. After surgery, the patient's power in the lower limbs began to improve, with significant recovery by discharge and complete resolution of bowel and bladder incontinence. This case highlights the diagnostic and therapeutic challenges of posterior epidural mass-like lesions in the lumbar spine, emphasizing the importance of prompt surgical intervention in restoring neurological function. The successful outcome underscores the significance of early diagnosis and intervention in such cases, ultimately improving the patient's quality of life.

## Introduction

Sequestration of a disc is described as the herniation of the disc, which perforates the posterior longitudinal ligament, ultimately leading to the migration of the disc fragments into the epidural space [1]. The disc usually migrates in the direction of the anterior epidural space, especially in the lateral, inferior, or superior sites. Thus, migration towards the posterior epidural disc is very rare and was first described by Lombardi in 1973 [2]. Up to 28.6% of all disc herniations are caused by intervertebral disc sequestration, wherein an extruded disc moves into the spinal canal, causing lumbar radiculopathy and cauda equina syndrome (CES) [3]. As commonly occurring in the lumbar region, it was further quoted as posterior epidural migration of lumbar intervertebral disc fragment (PEMLIF). Epidemiologically, males are more likely than females to develop posterior epidural migration of an extruded disc fragment, with a male-to-female ratio of roughly 4 to 1. This syndrome, which typically affects people in their middle years, is mistaken chiefly for lesions that take up posterior epidural space such as abscesses, tumors, cysts, and hematomas [4-6].

The rarity of this condition has resulted in a limited understanding of the root cause behind this condition. Furthermore, despite the potential for severe neurological impairment associated with PEMLIF, diagnosing and treating this condition remains quite challenging. By documenting a unique case of right-sided PEMLIF mimicking an epidural mass, this study aims to shed light on the atypical manifestations of this condition. The study seeks to contribute to the existing medical literature and assist healthcare professionals in effectively recognizing and treating PEMLIF. To the author’s best knowledge, this is the first case report documenting an unusual case of right-sided PEMLIF mimicking an epidural mass.

## Case presentation

Patient information

A 53-year-old man with a history of hypertension came to the Neurosciences Outpatient Department with a recent complaint of persistent lower back pain that has been bothering him for two years. The pain was radicular in nature and had an acute aggravation for the last five days, which impeded the patient's ability to ambulate. Furthermore, the patient described experiencing numbness in his lower limbs, urinary incontinence, and irregular bowel movements. Notably, the patient did not mention any previous instances of injury or swelling in the affected area.

Clinical findings

The clinical examination of the lower limbs indicated normal muscle bulk and tone. However, there was reduced power, graded at 4/5, in both hips. The power in the left knee was also noted as 5/5, whereas it was 3/5 in the right knee. Furthermore, the power was graded at 1/5 on dorsiflexion and 2/5 on plantarflexion, bilaterally in the ankles. The examination also revealed a positive straight leg test and reduced sensation along the L3 dermatome.

Diagnostic approach

**Figure 1:**
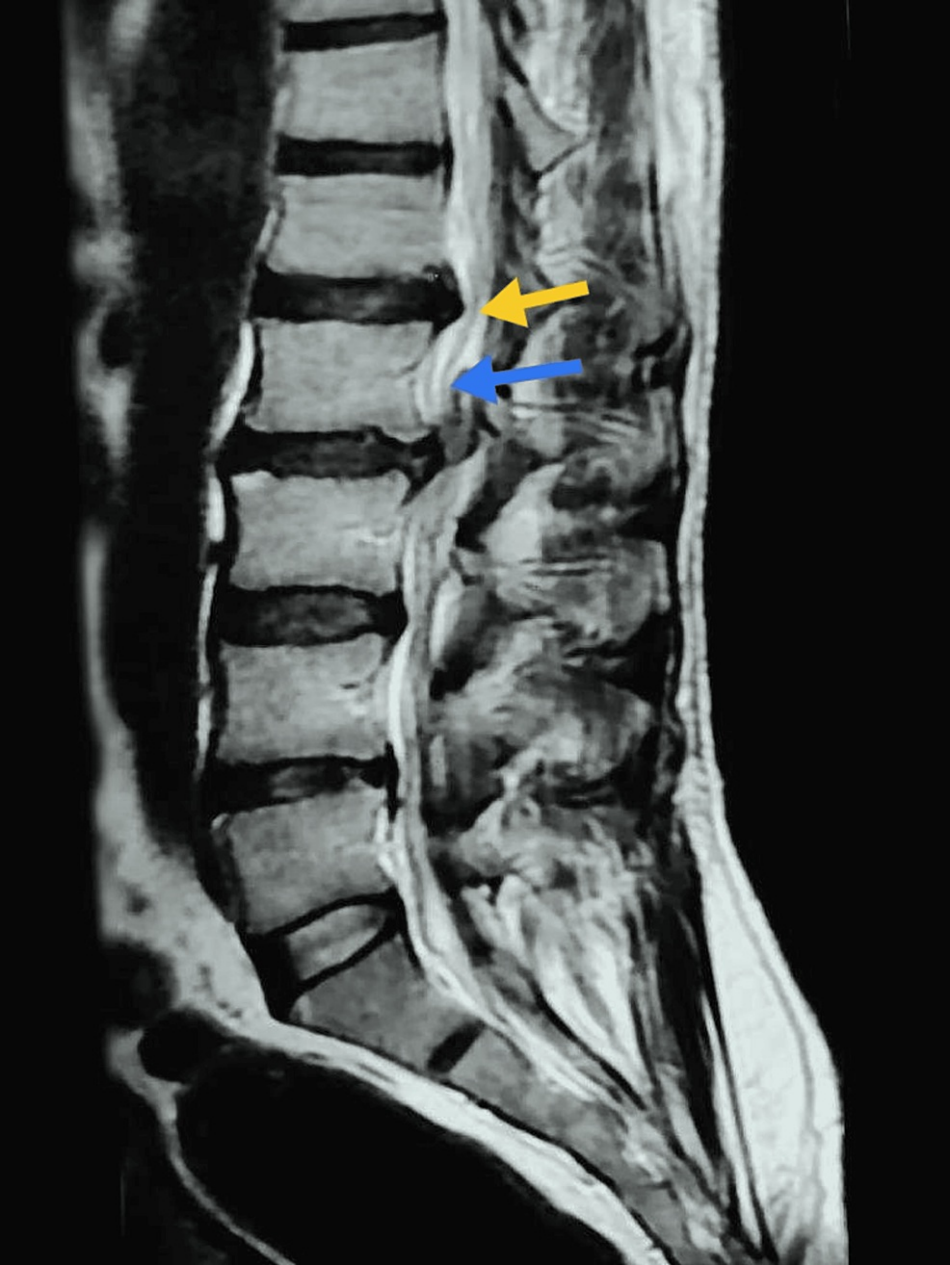
T2-weighted image of a sagittal view of the lumbar spine showing posteriorly migrated disc (shown by an yellow arrow) compressing the nerve root and thecal sac (shown by an blue arrow)

Following a magnetic resonance imaging (MRI) of the spine, the results revealed the presence of a posterior epidural mass measuring 3.5 cm×2 cm×1 cm at the L2-L3 level. This mass appeared isointense with the intervertebral disc on both T1- and T2-weighted images. Additionally, there was evidence of a disrupted outer annulus in the adjacent disc and tract-like enhancement extending into the right posterior epidural space (Figure 1).

Therapeutic intervention

**Figure 2:**
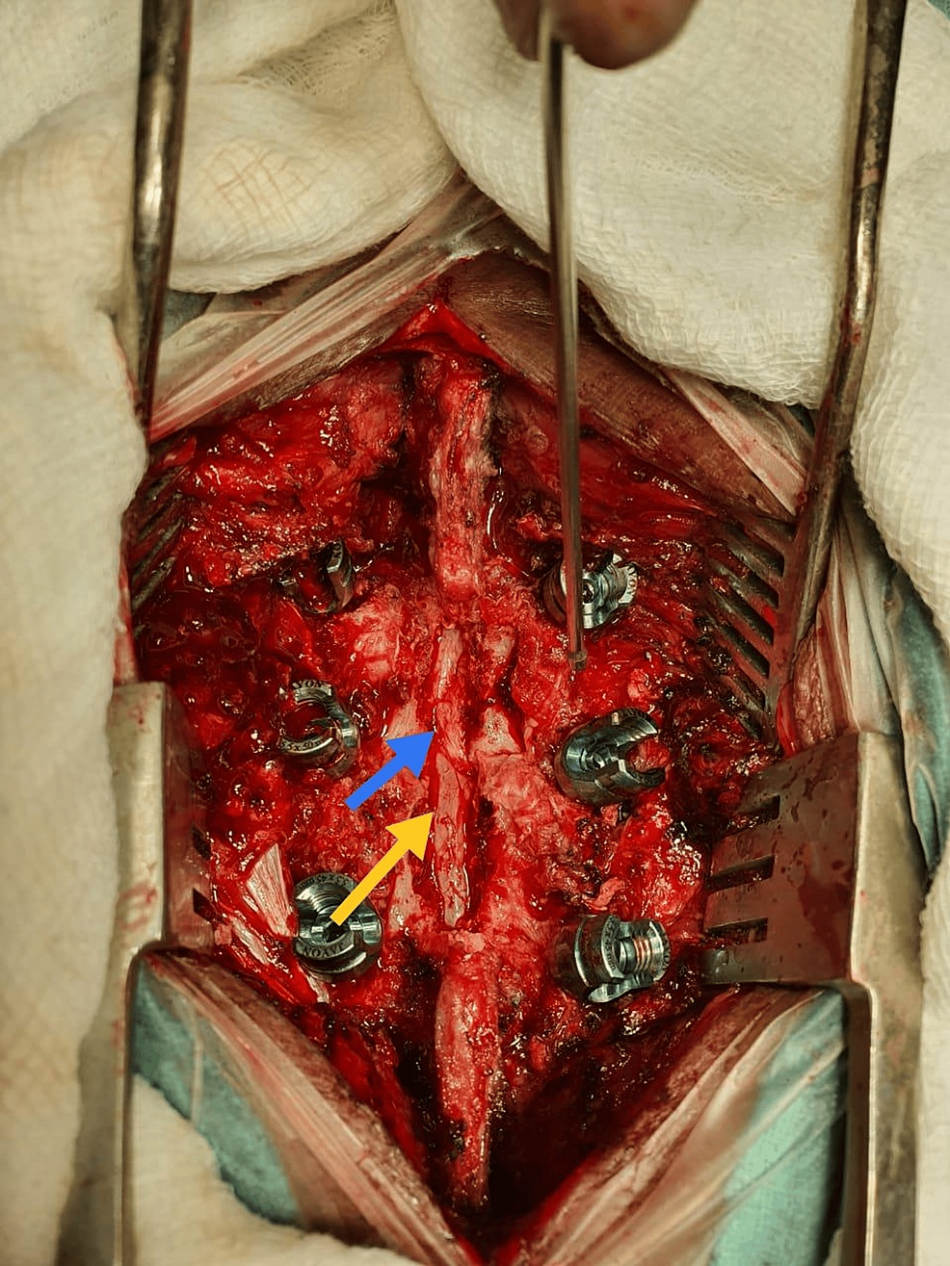
Intraoperative images showing thecal sac (shown by an yellow arrow) and posteriorly migrated disc seen just after removing lamina and spinous process (shown by an blue arrow)

The patient underwent an L3 laminectomy using a posterior approach. Following the visualization and removal of the ligamentum flavum, extruded disk fragments that resembled an epidural tumor were observed. These fragments were embedded in fibrous epidural tissue, causing thecal sac compression (Figure 2).

On histological examination, the diagnosis of PEMLIF was confirmed.

These fragments were removed with great precision, and the L2-L3 space was examined. A rupture in the posterior longitudinal ligament was discovered, and L2-L3 discectomy was carried out.

Outcome and follow-up

On postoperative day one, the patient began to regain power in the lower limbs, recorded 3/5 bilaterally. On discharge, the patient regained the power of 4/5 bilaterally with no bowel and bladder incontinence. At three months of follow-up, the patient could walk without support and had no bowel and bladder involvement.

## Discussion

The above-discussed case is extremely rare and was initially described in 1973 by Vincenzo Lombardi [2]. While the pathogenesis is not well-known, the most probable theory is that the herniated disc usually follows the pathway of least resistance, i.e., posterior or poster-lateral migration to the anterior epidural space of the spinal cord. This is because of the anatomical limitations of the posterior lateral ligaments, middle septum, and lateral membranes [7]. Furthermore, the midline and lateral ligaments of Hoffman, epidural vessels, fat, and nerve root also contribute to the limitation provided by the posterior lateral ligaments [8]. Due to these barriers, the disc seldom migrates to the posterior epidural space and can be misdiagnosed as other more probable lesions-like cysts, hematomas, abscesses, and malignancies.

Clinical signs of free fragment migration are usually associated with radicular pain, but the posterior migration of fragments can also lead to CES [3]. The clinical features of our patient, including radiculopathy and muscle power deficits in the affected limb, were recorded for a shorter duration of time pertaining to this rare condition [1,6]. Our patient presented with chronic back pain, which was radicular in nature and aggravated acutely.

The preferred method for diagnosing this condition is a lumbar spine MRI scan enhanced with gadolinium, considered the gold standard. A study conducted in the recent past reported a 39.2% incidence of posterior migrations of the disc at the L3-L4 level due to the migration-conducive anatomy at that level [3]. On the other hand, our patient is observed to have this lesion at the L2-L3 level. This can be attributed to almost similar anatomy at adjacent disc space levels.

The scan of our lesion showed a hypointense T1-weighted image and around 80% of cases demonstrated a hyperintense T2 scan [9]. This hyperintensity is attributed to an increased water content of the herniated fragment. On the other hand, the remaining 20% of cases showed isointensity compared to the disc on the T2-weighted image. Our case had similar findings on the scans. Tumors, more often, are uniformly enhanced on the scans. Therefore, a sequestrated disc was most likely the preoperative diagnosis in our situation, but an epidural tumor was not completely ruled out.

Regarding the other differentials considered, facet cysts have peculiar signal intensity on scans with distinct anatomical associations. An epidural abscess may present findings similar to PEMLIF, but the absence of hallmark signs and symptoms of infectious etiology rules out this diagnosis. Hematoma usually presents with isointense or hyperintense T1-weighted images with a history of trauma. The migrated fragments often show no continuity with the disc space of origin. On the other hand, hematomas maintain this continuity [10]. Many cases have reported a left-sided herniation [1,11], whereas our report is the first documented report on right-sided herniation.

## Conclusions

Posterior epidural migration of lumbar disc fragments is an exceedingly rare condition. It may manifest with symptoms such as radicular compression, lower back pain, or even the complex presentation of CES. Rim augmentation, which can resemble the symptoms of an intraspinal extradural tumor, makes it difficult to make a preoperative diagnosis. It is believed that an inflammatory reaction, manifested by the growth of vascular granulation tissue around the disc mass, is responsible for the peripheral enhancement of the disc fragment seen in contrast-enhanced imaging investigations.

To arrive at an accurate diagnosis of PEMLIF, considering factors like the patient's medical history, a detailed analysis of MRI scans to identify similarities in signal intensities between the disc fragment and the adjacent intervertebral disc and peripheral rim enhancement is crucial for the precise diagnosis of this rare spinal condition.
